# Serotypes and Clonal Composition of Streptococcus pneumoniae Isolates Causing IPD in Children and Adults in Catalonia before 2013 to 2015 and after 2017 to 2019 Systematic Introduction of PCV13

**DOI:** 10.1128/Spectrum.01150-21

**Published:** 2021-12-08

**Authors:** A. Redin, P. Ciruela, M. F. de Sevilla, F. Gomez-Bertomeu, S. Gonzalez-Peris, M. A. Benitez, G. Trujillo, A. Diaz, E. Jou, C. Izquierdo, M. O. Perez-Moreno, F. Moraga-Llop, M. Olsina, B. Vinado, E. Sanfeliu, A. Garcia, S. Gonzalez-di Lauro, J. J. Garcia-Garcia, A. Dominguez, R. Sa-Leao, C. Muñoz-Almagro

**Affiliations:** a Institut de Recerca Sant Joan de Deu, Hospital Sant Joan de Deu, Barcelona, Spain; b Departament de Medicina, Universistat Internacional de Catalunya, Barcelona, Spain; c CIBER de Epidemiologia y Salud Publica (CIBERESP), Instituto de Salud Carlos III, Madrid, Spain; d Agencia de Salut Publica de Catalunya (ASPCAT), Generalitat de Catalunya, Barcelona, Spain; e Hospital Universitari Joan XXIII, Tarragona, Spain; f Hospital Universitari Vall d’Hebron, Barcelona, Spain; g Hospital comarcal Alt Penedes, Barcelona, Spain; h Xarxa Assitencial Universitària de Manresa, Manresa, Spain; i Hospital de Nens, Barcelona, Spain; j Hospital Residència Sant Camil, Sant Pere de Ribes, Spain; k Consorci del Laboratori Intercomarcal de l'Alt Penedès, l'Anoia i el Garraf, Barcelona, Spain; l Hospital de Tortosa Verge de la Cinta, Tortosa, Spain; m Hospital Universitari General de Catalunya, Sant Cugat, Spain; n Hospital Sant Jaume d'Olot, Olot, Spain; o Hospital Universitario de Igualada, Igualada, Spain; p Hospital de Sant Joan Despí, Sant Joan Despí, Spain; q Departament de Medicina, Universitat de Barcelona, Barcelona, Spain; r Instituto de Tecnologia Química e Biológica, Oeiras, Portugal; University of Georgia

**Keywords:** *Streptococcus pneumoniae*, serotypes, clones, pneumococcal conjugate vaccines, invasive pneumococcal disease

## Abstract

The goal of this study was to investigate the distribution of serotypes and clonal composition of Streptococcus pneumoniae isolates causing invasive pneumococcal disease (IPD) in Catalonia, before and after systematic introduction of PCV13. Pneumococcal strains isolated from normally sterile sites obtained from patients of all ages with IPD received between 2013 and 2019 from 25 health centers of Catalonia were included. Two study periods were defined: presystematic vaccination period (2013 and 2015) and systematic vaccination period (SVP) (2017 to 2019). A total of 2,303 isolates were analyzed. In the SVP, there was a significant decrease in the incidence of IPD cases in children 5 to 17 years old (relative risk [RR] 0.61; 95% confidence interval [CI] 0.38 to 0.99), while there was a significant increase in the incidence of IPD cases in 18- to 64-year-old adults (RR 1.33; 95% CI 1.16 to 1.52) and adults over 65 years old (RR 1.23; 95% CI 1.09 to 1.38). Serotype 8 was the major emerging serotype in all age groups except in 5- to 17-year-old children. In children younger than 5 years old, the main serotypes in SVP were 24F, 15A, and 3, while in adults older than 65 years they were serotypes 3, 8, and 12F. A significant decrease in the proportions of clonal complexes CC156, CC191, and ST306 and an increase in those of CC180, CC53, and CC404 were observed. A steady decrease in the incidence of IPD caused by PCV13 serotypes indicates the importance and impact of systematic vaccination. The increase of non-PCV13 serotypes highlights the need to expand serotype coverage in future vaccines and rethink vaccination programs for older adults.

**IMPORTANCE** We found that with the incorporation of the PCV13 vaccine, the numbers of IPD cases caused by serotypes included in this vaccine decreased in all of the age groups. Still, there was an unforeseen increase of the serotypes not included in this vaccine causing IPD, especially in the >65-year-old group. Moreover, a significant increase of serotype 3 included in the vaccine has been observed; this event has been reported by other researchers. These facts call for the incorporation of more serotypes in future vaccines and a more thorough surveillance of the dynamics of this microorganism.

## INTRODUCTION

Streptococcus pneumoniae is a microorganism that can be found colonizing the human nasopharynx without causing any disease. However, in certain circumstances it can act as a pathogen, causing invasive diseases such as meningitis, sepsis, and pneumonia (1–4) accounting for a significant number of mortality and morbidity cases worldwide (4–7), particularly in children less than 5 years old and adults over 65 years (8–11).

The polysaccharide capsule of S. pneumoniae is a major virulence factor (12). Currently, more than 100 serotypes have been described (13, 14); however, due to the complexity of conjugate vaccines’ manufacturing, only a few of them are in the commercialized pneumococcal conjugate vaccines (PCVs).

The first available vaccine against this microorganism was the pneumococcal polysaccharide vaccine PPSV23, commercialized in Spain in 1983. However, PPSV23 was found not to be very effective in the children’s population, and in the adult population the results were found to be discordant (15). For this reason, the PPSV23 has been recommended only in the older adults and immunocompromised patients (16). In 2017, it was estimated that 63.1% of adults 65 to 79 years and 81.2% of those ≥80 years were vaccinated with PPSV23 in Catalonia (17). In 2001, a 7-valent pneumococcal conjugate vaccine (PCV7) targeting seven serotypes (4, 6B, 9V, 14, 18C, 19F, and 23F), highly effective in children (18), became commercially available in Spain. Before this first PCV was introduced, only a few serotypes accounted for more than 60% of invasive pneumococcal disease (IPD) cases in Catalonia in the pediatric population (19). Overall, vaccination of children with PCV7 reduced significantly the IPD burden worldwide in vaccinated and nonvaccinated populations due to the direct and indirect action (herd protection) of the vaccine (20). In addition, a significant increase of nonvaccine serotypes was observed (8).

Novel vaccines with additional serotypes were developed (21–23): PCV10 (PCV7 serotypes plus 1, 5, and 7F) and PCV13 vaccines (PCV10 serotypes plus 3, 6A, and 19A) (22, 24). Both were introduced in Spain in 2010. In Catalonia, a region of Spain, these PCVs were recommended by pediatricians since commercialization but parents had to pay the full cost of vaccine shots until July 2016 when PCV13 was included on the systematic vaccination schedule and administered for free in children at 2, 4, and 11 months of age. In 2017 and 2018, a national coverage of vaccination in children younger than 12 months was estimated to be 95% and 97.7%, respectively (https://www.mscbs.gob.es/profesionales/saludPublica/prevPromocion/vacunaciones/calendario-y-coberturas/coberturas/home.htm).

As observed in PCV7 and PCV10, incorporation of the PCV13 vaccine appeared to be very effective in decreasing the number of IPD cases caused by serotypes included in the vaccine in all of the age groups (22). Still, there was an unforeseen increase of non-PCV13 serotypes causing IPD. However, vaccine failures against serotype 3 have been reported (24, 25). In addition, non-PCV13 serotypes are an important cause of IPD in adults older than 65 years (21, 26). These facts call for the incorporation of more serotypes in future vaccines, apart from the PCV15 vaccine (PCV13 plus 22F and 33F) and PCV20 vaccine (PCV13 plus 8, 10A, 11A, 12F, 15B, 22F, 33F) which have currently been approved by the FDA (25, 27).

The objective of this study is to update the serotype and clonal distribution of S. pneumoniae causing IPD disease in Catalonia, Spain, comparing two periods: presystematic vaccination period (PSVP) (2013 to 2015) and systematic vaccination period (SVP) (2017 to 2019) in four age groups (<5-year-old children, 5- to 17-year-old children, 18- to 64-year-old adults, and ≥65-year-old adults). Data of PSVP have been published previously (28). Our aim was to describe relevant changes that may address the need of adopting new conjugate vaccines targeting additional serotypes.

## RESULTS

Serotype and clonal data taken from the presystematic vaccination period (PSVP, 2013 to 2015) and systematic vaccination period (SVP, 2017 to 2019) included a total of 2,303 episodes of IPD, which occurred in 2,258 patients (45 recurrent episodes). A total of 1,048 episodes were detected in the PSVP and 1,255 in the SVP. Over half of the episodes occurred in the male population (1,358; 58.9%). The patients’ median age was 57 years (range 1 day to 100 years). A total of 286 (12.4%) of the strains were isolated from <5-year-old children, 71 (3.1%) were isolated in 5- to 17-year-old children, 831 (36.1%) in 18- to 64-year-old adults, and 1,115 (48.4%) in 65-year-old and older adults. Most of the cultures were obtained from blood samples (92.7%), cerebrospinal fluid (CSF) (69; 3%), and pleural fluid (62; 2.7%).

### Proportion and incidence rates of IPD episodes in presystematic and systematic vaccine periods.

After systematic introduction of the PCV13 vaccine, a significant decrease of overall IPD cases was observed for all ages (RR 1.19; 95% confidence interval [CI] 1.1 to 1.30). A significant decrease was perceived in children 5 to 17 years old (RR 0.61; 95% CI 0.38 to 0.99), but no significant changes were observed in younger children (RR 0.95; 95% CI 0.75 to 1.20). In adults, a significant increase of IPD cases was observed in those between 18 and 64 years (RR 1.33, 95% CI 1.16 to 1.52) and in the ≥65-year-old group (RR 1.23, 95% CI 1.09 to 1.38).

IPD incidence rates due to PCV13 serotypes decreased significantly in younger children (RR 0.58; 95% CI 0.39 to 0.87), older children (RR 0.41; 95% CI 0.21 to 0.78), and ≥65-year-old adults (RR 0.80; 95% CI 0.65 to 0.97). No significant variations in the incidence rate of IPD caused by the vaccine serotypes were observed in the younger adults (RR 0.96; 95% CI 0.76 to 1.21). Overall, there was a significant decrease of IPD cases caused by PCV13 serotypes (RR 0.79; 95% CI 0.69 to 0.91).

Contrariwise, IPD caused by non-PCV13 serotypes exhibited an overall significant increase (RR 1.50; 95% CI 1.36 to 1.67). For 18- to 64-year-old adults (RR 1.58; 95% CI 1.33 to 1.88) and adults 65 and above years old (RR 1.54; 95% CI 1.33 to 1.79), a significant increase was observed. For <5-year-old (RR 1.25; 95% CI 0.93 to 1.67) and 5- to 17-year-old children (RR 1.09; 95% CI 0.52 to 2.29), no significant differences between PSVP and SVP were detected for the nonvaccine serotypes.

Among both periods, the highest incidence was observed in seniors, followed by younger children. Proportion and incidence rates of IPD episodes in PSVP and SVP according to group of age can be found in Table 1. In addition, Fig. S1 shows IRRs per year estimated by comparing observed incidence (in each of the years 2014 to 2019) to reference (incidence in 2013) for each age-serotype group.

**TABLE 1:** Cases and incidence rates of IPD in PSVP and SVP^*a*^

IPD cause	Age group	Cases in PSVP (%)	Cases in SVP (%)	*P* value	IR in PSVP	IR in SVP	IRR (95% CI)	*P* value	Adjusted *P* value^*b*^
All serotypes									
	<5	153 (14.6)	133 (10.6)		20.94	19.88	0.95 (0.75–1.20)	0.331	0.382
	5–17	43 (4.1)	28 (2.2)		2.46	1.52	0.61 (0.38–0.99)	0.022	0.03
	18–64	360 (34.4)	471 (37.5)		7.15	9.50	1.33 (1.16–1.52)	<0.001	0.002
	≥65	492 (46.9)	623 (49.6)		30.97	37.98	1.23 (1.09–1.38)	<0.001	0.002
	All ages	1,048 (100)	1,255 (100)		11.52	13.77	1.19 (1.1–1.30)	<0.001	0.002
PCV13 serotypes									
	<5	68 (44.4)	36 (27.1)	<0.001	9.31	5.38	0.58 (0.39–0.87)	0.003	0.005
	5–17	30 (69.8)	13 (46.4)	<0.001	1.72	0.70	0.41 (0.21–0.78)	0.002	0.004
	18–64	148 (41.1)	140 (29.7)	0.016	2.94	2.82	0.96 (0.76–1.21)	0.367	0.393
	≥65	208 (42.3)	171 (27.4)	<0.001	13.09	10.42	0.80 (0.65–0.97)	0.013	0.019
	All ages	454 (100)	360 (100)	<0.001	4.99	3.95	0.79 (0.69–0.91)	<0.001	0.002
Non-PCV13 serotypes									
	<5	85 (55.6)	97 (72.9)	0.367	10.63	14.50	1.25 (0.93–1.67)	0.069	0.086
	5–17	13 (30.2)	15 (53.6)	0.459	0.75	0.81	1.09 (0.52–2.29)	0.413	0.413
	18–64	212 (58.9)	331 (70.3)	<0.001	4.21	6.67	1.58 (1.33–1.88)	<0.001	0.002
	≥65	284 (57.7)	452 (72.6)	<0.001	17.87	27.55	1.54 (1.33–1.79)	<0.001	0.002
	All ages	594 (100)	895 (100)	<0.001	6.53	9.82	1.50 (1.36–1.67)	<0.001	0.002

a IPD, Invasive pneumococcal disease; PSVP, presystematic vaccination period; SVP, systematic vaccination period; IR, incidence rate per 100,000 people per year; IRR, incidence rate ratio; CI, confidence interval. Incidence: episodes per 100,000 people per year living in the reference area of 25 health centers according to the data from “Catalonian Institute of Statistics” (www.idescat.net).

b Adjusted *P* values were calculated using FDR. *P* < 0.05 (chi-square test).

### Serotype distribution and incidence rates according to serotypes in PSVP and SVP.

**FIG 1:**
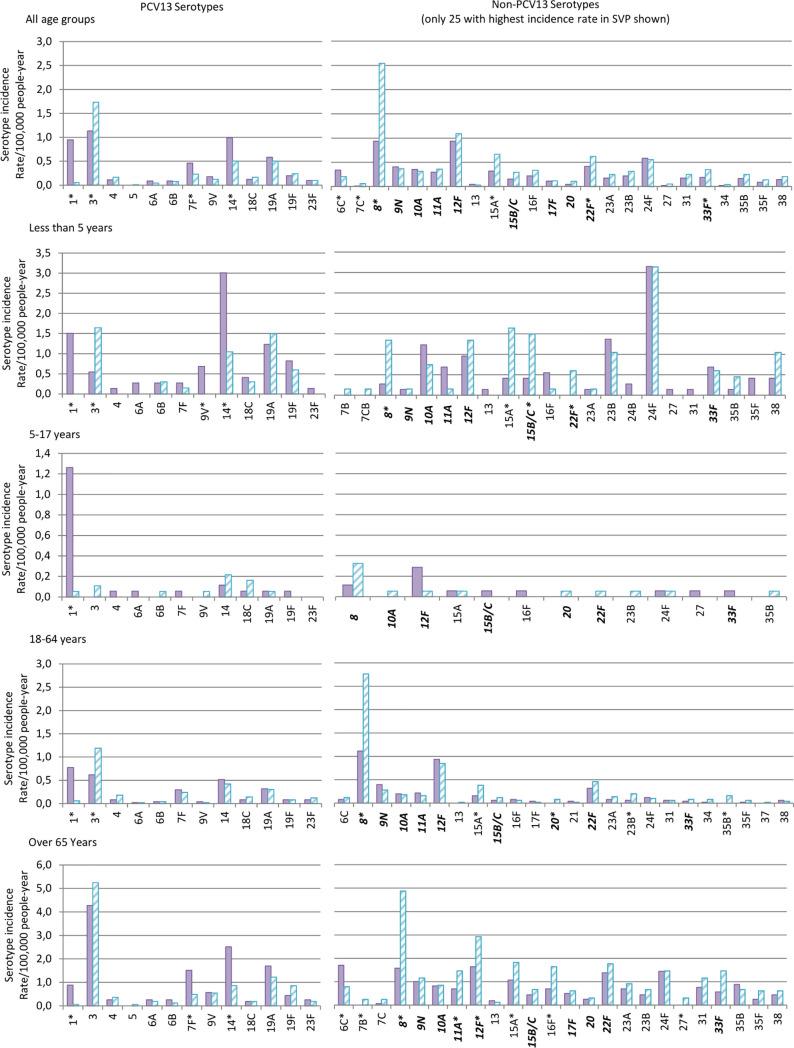
Serotype distribution and incidence rate per 100,000 people per year of invasive serotypes during the study period and stratified by age groups. PSVP, presystematic vaccination period; SVP, systematic vaccination period. Filled bars represent PSVP, while stripped ones represent SVP. *, *P* < 0.05 (chi-square test). Patients have been grouped in four different age groups. Non-PCV13 serotypes marked in cursive and bold are the ones included in the PPSV23 vaccine.

PCV13 serotypes accounted for 43% (454) of the IPD cases in PSVP, whereas in SVP they accounted for 29% (361) of the IPD cases. In all age groups, the non-PCV13 serotypes were the predominant serotypes causing IPD cases in the systematic period, with the exception of older children, which showed similar IPD rates among PCV13 versus non-PCV13 serotypes. IPD rates by serotype group for each of the different age groups can be found in Fig. 1 and Table S2.

Overall, the incidence of three (1, 7F, and 14) of the PCV13 serotypes decreased significantly in the systematic period, serotype 1 being the one that suffered a more drastic decrease, nearly disappearing during the systematic period. Incidence of serotype 3 increased significantly from the PSVP to the SVP period, while no changes were observed for serotype 19A. The other PCV13 serotypes practically disappeared since PSVP. Concerning non-PCV13 serotypes, serotypes 8 and 12F (both of them included in PPSV23) were the most frequent, together with serotype 3, for most of the IPD cases in the SVP period (Fig. 1).

The observed PCV13 serotype trends were quite similar between the different age groups, with some differences in specific serotypes. Among the SVP period, for the <5-year-old children age group, the most frequent serotypes observed were 3 and 19A with significant increase for serotype 3, while in the 5- to 17-year-old children, the predominant serotype observed was 14 without any significant variation compared to the PSVP period. Both 18- to 64-year-old and ≥65-year-old adults experienced a rise in the number of IPD cases caused by serotype 3 in the SVP, placing it as the most common serotype causing this disease.

Incidence of non-PCV13 serotypes varied substantially between age groups. In <5-year-old children, the most frequent serotypes in the systematic period were 24F and 15A, with significant incidence increase for 15A, followed by serotypes 8, 22F, and 15B/C. In 5- to 17-year-old children, serotype 8 was the most common serotype in the systematic vaccination period, versus serotype 12F, which was the dominant serotype in the presystematic vaccination period, with no significant incidence variation. Adults 18 to 64 years old and seniors ≥65 years old shared the same predominant serotypes for both PSVP and SVP periods. Of note, a significant increase of serotypes 8, 12F, and 11A, which are included in PPSV23 vaccine, were observed in adults older than 65.

Serotype variation along the years can be observed in Table S3.

**FIG 2:**
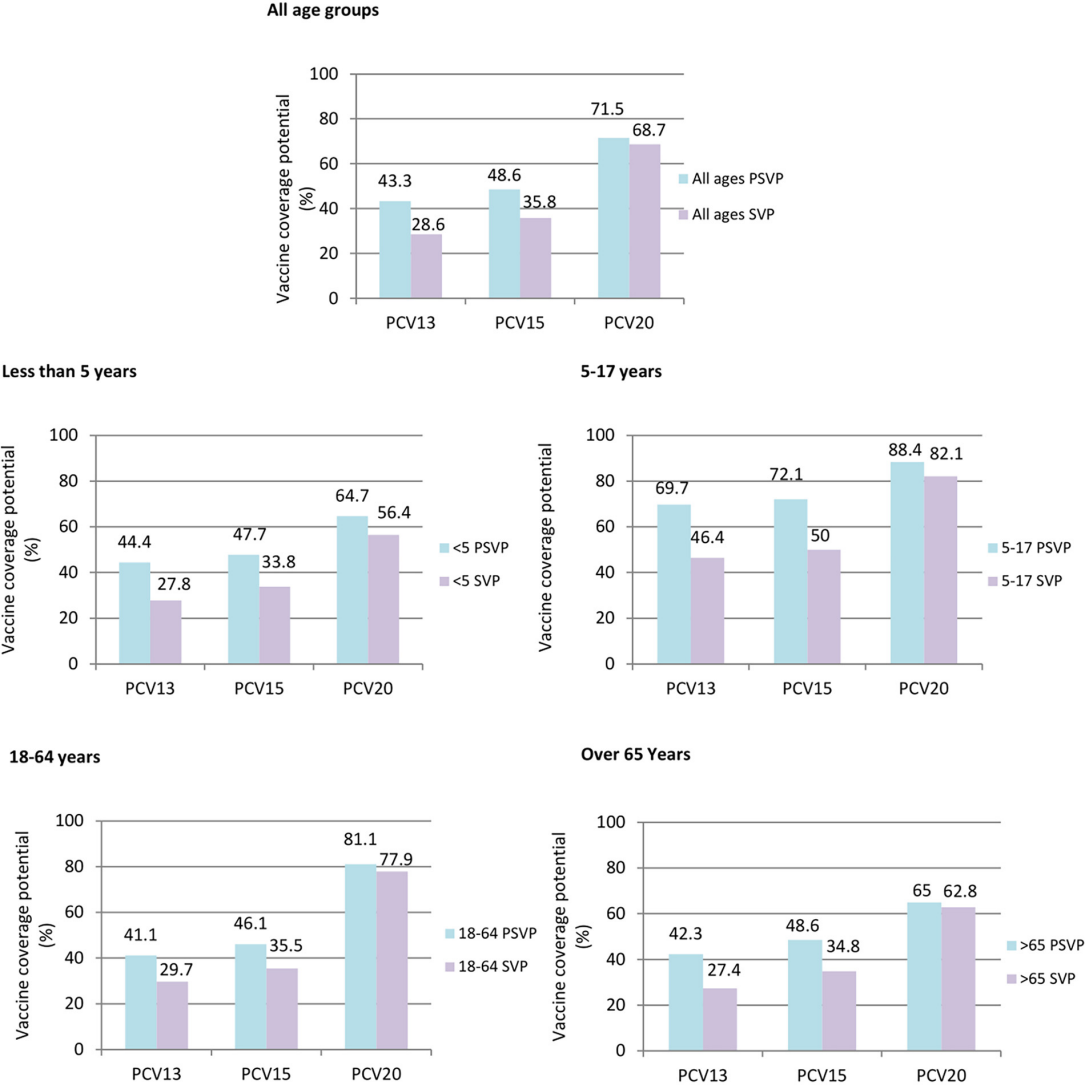
Potential coverage of PCV13 (serotypes 1, 3, 4, 5, 6A, 6B, 7F, 9V, 14, 18C, 19A, 19F, and 23F), PCV15 (PCV13 serotypes plus 22F and 33F), and PCV20 (PCV13 serotypes plus 8, 10A, 11A, 12F, 15B, 22F, and 33F) according to the observed proportion of the serotypes included in each of these vaccines for both study periods (PSVP and SVP) and stratified by age groups (<5, 5 to 17, 18-64, and >65). PSVP, presystematic vaccination period; SVP, systematic vaccination period.

Figure 2 shows the potential coverage of the vaccines PCV13, PCV15, and PCV20 according to the observed proportion of the serotypes included in each of these vaccines for both study periods and stratified by age groups.

### Clonal distribution of isolates.

**FIG 3:**
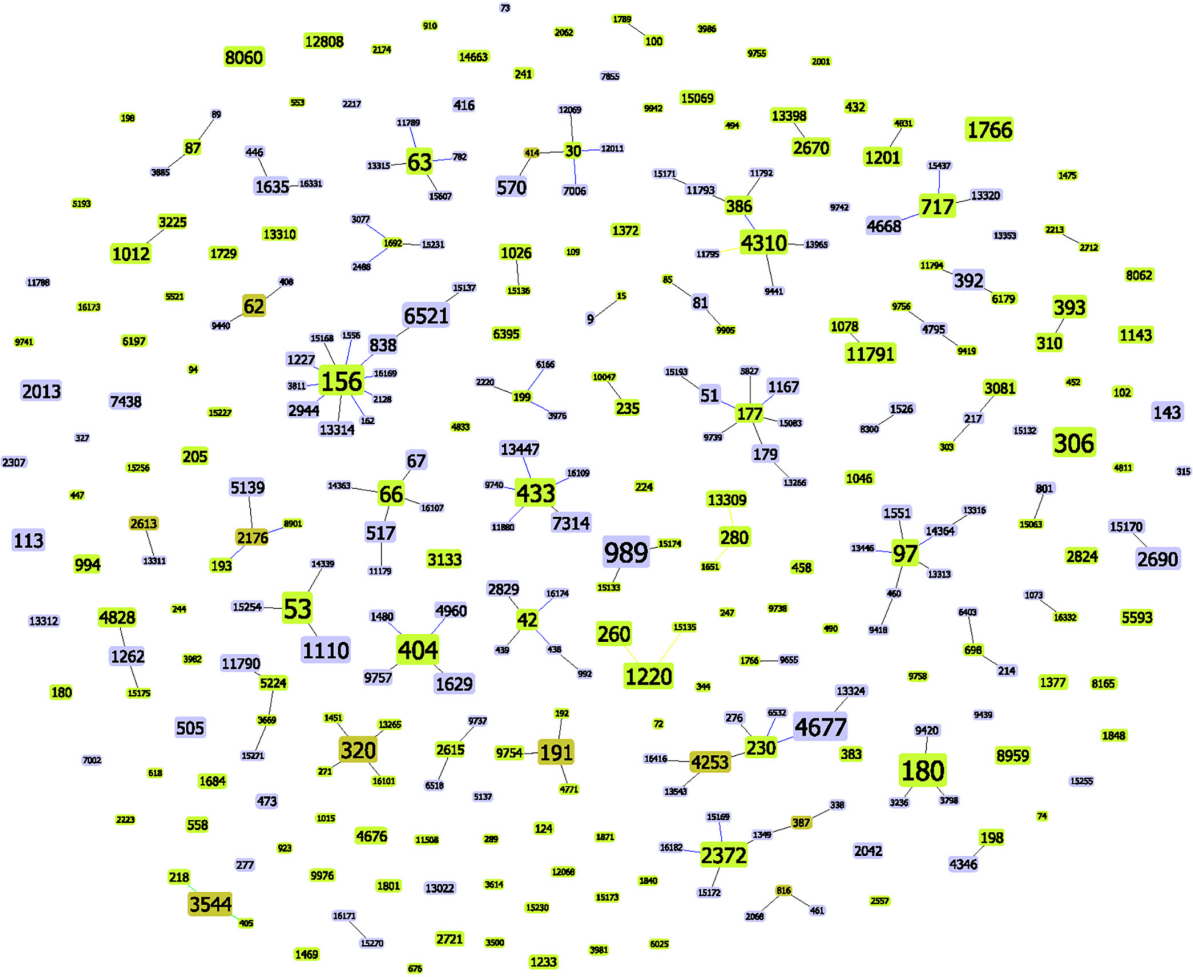
eBURST map of clonal complexes (CCs) and singletons found during the study period 2013 to 2019. Green nodes represent group founders; blue nodes represent common nodes. Black links drawn without recourse to tiebreak rules; blue links drawn using tiebreak rule 1 (number of single locus variants [SLVs]); green links drawn using tiebreak rule 2 (number of double locus variants [DLVs]); yellow links drawn using tiebreak rule 4 or 5 (frequency found on the data set and ST number). One link between group founder and an ST is considered an SLV. Two links between group founder and an ST is considered a DLV, and three links is a triple locus variant (TLV).

A total of 318 sequence types (ST) were identified among the 2,303 invasive strains isolated between 2013 and 2019 (Fig. 3). Taking into consideration that a clonal complex (CC) was defined as an ST sharing five out of seven allelic variants (double locus variants), around 63% of the ST were grouped in 53 different CCs accounting for 200 ST with a total of 1,871 isolates (81.2%), with one of them being a triple locus variant. The remaining 118 STs accounting for 432 isolates (18.8%) were singletons.

Comparing both periods, statistically significant decreases were detected in the proportions of certain CCs associated with PCV13 serotypes, such as CC156 associated mainly with serotype 14, ST306 with serotype 1, CC191 with serotype 7F, and CC386 with serotype 6C. Regarding CC320 (related to serotype 19A), no significant variations were observed. A significant increase was observed in CC180 related to serotype 3 and CC218 related to serotype 7F. When observing the CCs associated with mainly non-PCV13 serotypes, statistically significant increases in CC404 and CC53 were seen, both related to serotype 8. However, no significant changes were observed in CC989 and CC230 related to serotypes 12F and 24F, respectively. CCs with more than 28 isolates are detailed in Table 2.

**TABLE 2:** Clonal complexes (CCs) and singletons with more than 28 isolates for the study periods (2013 to 2019) and their associated serotypes for all age groups^*a*^

CC/ST	Serotype	No. cases in PSVP (%) *n* = 1,048	No. cases in SVP (%) *n* = 1,255	*P* value	Adjusted *P* value^*b*^
CC156		96 (9.2)	67 (5.3)	<0.001	0.003
	14	75 (78.1)	38 (56.7)		
	9V	7 (7.3)	4 (6)		
	11A	14 (14.6)	23 (34.3)		
	24F	0	1 (1.5)		
	15A	0	1 (1.5)		
CC180		57 (5.4)	117 (9.3)	<0.001	0.003
	3	57 (100)	117 (100)		
CC53		51 (4.9)	142 (11.3)	<0.001	0.003
	8	51 (100)	142 (100)		
CC230		52 (5)	52 (4.1)	0.173	0.232
	24F	48 (92.4)	48 (92.4)		
	19A	2 (3.8)	2 (3.8)		
	24B	2 (3.8)	0		
	15B/C	0	2 (3.8)		
ST306		80 (7.6)	4 (0.3)	<0.001	0.003
	1	77 (96.25)	4 (100)		
	NT	3 (3.75)	0		
CC404		30 (2.8)	80 (6.4%)	<0.001	0.003
	8	30 (100)	80 (100)		
CC191		36 (3.4)	2 (0.2)	<0.001	0.003
	7F	36 (100)	2 (100)		
CC320		22 (2.1)	24 (1.9)	0.375	0.396
	19A	22 (100)	23 (95.8)		
	19F	0	1 (4.2)		
CC989		83 (7.9)	87 (6.9)	0.183	0.232
	12F	82 (98.8)	87 (100)		
	8	1 (1.2)	0		
CC63		26 (2.5)	23 (1.8)	0.144	0.232
	15A	23 (88.5)	20 (87.1)		
	19F	1 (3.8)	0		
	14	2 (7.7)	1 (4.3)		
	23B	0	1 (4.3)		
	23F	0	1 (4.3)		
CC433		35 (3.3)	53 (4.2)	0.137	0.232
	22F	35 (100)	52 (98.1)		
	22FA	0	1 (1.9)		
CC260		39 (3.7)	33 (2.6)	0.068	0.144
	3	39 (100)	33 (100)		
CC66		34 (3.2)	35 (2.8)	0.262	0.311
	9N	34 (100)	33 (94.3)		
	9NL	0	2 (5.7)		
CC42		9 (0.9)	16 (1.3)	0.174	0.232
	23A	9 (100)	15 (93.8)		
	23B	0	1 (6.2)		
CC2372		20 (1.9)	20 (1.6)	0.283	0.317
	23B	19 (95)	20 (100)		
	23F	1 (5)	0		
CC97		30 (2.8)	28 (2.2)	0.169	0.232
	10A	29 (96.7)	28 (100)		
	6C	1 (3.3)	0		
CC386		30 (2.8)	16 (1.3)	0.003	0.008
	6C	25 (80)	15 (93.8)		
	6B	5 (16.7)	1 (6.2)		
CC218		8 (0.8)	20 (1.6)	0.036	0.086
	7F	5 (62.5)	19 (95)		
	12F	2 (25)	1 (5)		
	9N	1 (12.5)	0		
CC177		18 (1.7)	20 (1.6)	0.408	0.408
	19F	10 (55.6)	16 (80)		
	19A	3 (16.7)	0		
	24F	5 (27.8)	1 (5)		
	23B	0	1 (5)		
	7C	0	2 (10)		

a CC, clonal complex (eBURST); ST, sequence type; PSVP, presystematic vaccination period; SVP, systematic vaccination period.

b Adjusted *P* value*s* were calculated using FDR %, serotype proportion compared with the number of clones in each study period. *P* < 0.05 (Chi-square test), clone proportion compared with the total number of episodes registered in PSVP (1,048) and SVP (1,255).

Concerning the clonal distribution across the different age groups for both periods (see Table S4), some differences were observed. In the younger children group, the most common clones were CC156, CC230, and ST306 for the PSVP period, whereas in the SVP period there was a predominance of CC230, CC989, CC180, CC156, and CC2372. In older children, the most frequent clone in PSVP was ST306 versus CC53 and CC156 in the SVP period. For the 18- to 64-year-old adults, four major clones were observed: CC989, ST306, CC53, and CC156 in PSVP versus CC53, CC404, CC180, and CC989 in SVP. Last, in the older adult group, the main clones for both PSVP and SVP were CC180, CC156, and CC989, with the exception of CC260 in PSVP and CC53 in SVP.

Half of the clonal complexes were associated with more than one serotype (50.9%), while most singletons were associated with a single serotype (93.2%). For example, all isolates associated with CC180 (*n* = 174), CC260 (*n* = 72), CC191 (*n* = 38), CC53 (*n* = 193), CC404 (*n* = 110), and ST306 (*n* = 81) were exclusively of serotypes 3, 3, 7F, 8, 8, and 1, respectively. Most (99.4%, *n* = 169) isolates of CC989 were of serotype 12F. Still, Table 2 shows that most CCs were associated with two or more serotypes. Of note, CC156, CC230, CC63, and CC177 were associated with more than five serotypes.

Changes in the serotype associated most frequently with a given CC were noted after the introduction of the PCV13 vaccine, although most CCs retain the dominant serotype. For example, in the PSVP, period CC156 was associated mainly with serotype 14 (78.1%), while during the SVP period, almost half of the isolates in this CC were of other serotypes; in particular, serotype 11A accounted for 34.3% of isolates (*P* < 0.001). On the other hand, a significant increase in the proportion of serotype 7F within isolates of CC218 was observed (from 62.5% to 95%, *P* < 0.006).

Table S5 shows the ST variation along the years.

## DISCUSSION

In this study, we have analyzed the incidence, the serotype distribution, and the clonal composition of invasive pneumococcal strains causing IPD in two different periods (SVP and PSVP) after the introduction of the PCV13 vaccine in 25 centers across Catalonia, Spain.

A significant decline of the number of IPD cases caused by vaccine serotypes was observed in all age groups, particularly in the young and older children’s groups, calling for a possible herd protection phenomenon. In fact, an overall decrease in the number of invasive cases caused by the most prevalent serotypes—1, 7F, and 14, all considered to be primary pathogens with high invasive disease potential (29, 30)—was observed in SVP. A decrease of these serotypes after PCV13 introduction has also been observed in other countries (31, 32) and is suggestive of vaccine effectiveness.

A previous study from our group (28) reported persistence of serotype 14 related to CC156; however, in the present study, a significant decrease of serotype 14, mainly in the <5-year-old children and ≥65-year-old adults, was observed. Interestingly, while serotype 14 decreased, serotype 11A related to ST6521, a double locus variant included in CC156, experienced a modest increase that could be associated with capsular switching (33). Monitoring of the evolution of these serotypes related to CC156 seems relevant for future vaccines. Concerning serotype 3, a significant increase was observed, placing it as one of the most prevalent serotypes causing IPD in young children, young adults, and seniors. This is worrisome, as it appears that the numbers of cases are increasing compared to those in our previous study. Moreover, this phenomenon has also been observed by other authors (34, 35). Despite it being included in the PCV13 vaccine, serotype 3 has been associated with vaccine failure, but unfortunately there is little knowledge about why this occurs. Some studies attribute this occurrence to its thick, noncovalently linked polysaccharide capsule (36, 37). Other studies highlight the importance of having high concentrations of anti-capsular antibody to ensure protection against serotype 3 (38). Additionally, changes in the genomic content of serotype 3 associated with clonal complex 180 have been observed in England and Wales (39), which could be responsible for the emergence of clade II, currently responsible for 50% of serotype 3 isolates causing IPD. Further investigation is needed in order to understand the impact it may have in vaccine effectiveness.

A significant decrease in the number of episodes causing IPD was observed in the older children’s group (40), while in the younger adults’ and seniors’ groups the incidence of IPD cases increased significantly. Overall, a general declining trend of serotypes included in the PCV13 vaccine was observed in all age groups, in contrast with what was observed for the non-PCV13 serotypes, of which number of cases increased. The decrease in the number of IPD cases caused by PCV13 is reassuring, as it confirms the success of the PCV13 vaccine. However, attention must be paid now to those serotypes not included in the vaccine, as their incidence is increasing and they are taking the lead as the major serotypes causing IPD (41–43). This serotype replacement phenomenon is more remarkable in adults, where the main leaders are now serotypes 8 and 12F, as observed in Fig. 1 and Table 1. Interestingly, in the younger children’s group, the main non-PCV13 serotypes causing disease are 24F and 15A, as observed in other studies (44, 45). Of note, these serotypes are not included in the new vaccines.

Concerning the non-PCV13 serotypes that are currently causing the majority of IPD cases, such as 8 and 12F, they are regarded as having a high invasive disease potential, as seen in our previous study (28). The increase in the number of disease episodes caused by these serotypes was correlated with the emergence of CC53, CC404, and CC989. In the SVP period, both CC53 and CC404 experienced a significant increase in the number of cases related to serotype 8, whereas CC989 related to serotype 12F did not experience any significant changes, as seen in Table 2. These tendencies have also been observed in other studies (34, 46, 47), with an increase in the circulation of CC53 in Canada (48) and CC989 in Israel (49). Fortunately, these serotypes will be included in the PCV20 vaccine which is currently under development (50) (https://clinicaltrials.gov/ct2/show/NCT03760146). Concerning the PPSV23 vaccine, the significant increase of serotypes 8, 11A, and 12F in ≥65-year-old adults despite the high rates of PPSV23 use in this population could suggest a limited effect in the protection against IPD disease (17).

In the <5-year-old group, serotype 24F remains the outstanding leader; however, a significant increase in the number of IPD cases caused by serotype 15A was also observed. Unfortunately, neither of these two serotypes will be included in the new conjugate vaccines PCV15 (PCV13 plus 22F and 33F) or PCV20 (PCV13 plus 8, 10A, 11A, 12F, 15B, 22F, 33F). Serotype 24F was associated mainly with CC230, which has remained rather stable between PSVP versus SVP, and serotype 15A was related to CC63, which also remained stable between periods. Increase of serotype 15A has also been observed in other countries, such as the United Kingdom (51), Germany (52), and Japan (53). Nonetheless, the emergence of this serotype and the persistence of serotype 24 mark the importance of including these two serotypes in future vaccines.

Another noticeable event observed in this study was the significant increase of CC218 related to serotype 7F. This is very interesting, as a significant decrease of this serotype in the SVP period was observed; however, this decrease was related mostly to CC191. The current surge of CC218 poses a concern and calls for future surveillance of this occurrence. Overall, changes in CC prevalence are not considered to simply reflect the changes in serotype prevalence.

This study shows changes in the predominant serotypes and clones after PCV13 introduction. Little is known, however, about the causes leading some serotypes or clones to be successful in their dissemination whereas others disappear (54). Even though it is evident that future vaccines should cover more serotypes, the limitations of the production of PCV vaccines should be taken into account. First of all, they are expensive and quite complex to produce (55). Moreover, increasing the number of serotypes in each vaccine further increases the price. These limitations call for a need to produce new types of pneumococcal vaccines that are non-serotype-dependent. These new promising pneumococcal vaccines, which are in clinical trials, should be able to cover a broad range of serotypes, induce systemic and mucosal immunity, and protect against invasive disease and pneumococcal carriage (56).

This study has several limitations. First of all, our findings may not be representative of the whole Catalonian population, as we included only the health facilities that consistently sent their samples to the reference laboratory to avoid over- or underrepresentation across different calendar years. By doing this, we are confident of the obtained yearly representation. Moreover, the results obtained in this study display an overall consistency in comparison with the results collected in our previous study period (2011 to 2016) in Catalonia (28). Our study population comprises 62% of the Catalan children (younger than 18 years), whereas the adult population (older than 18 years) had a representation of 36%. To avoid overrepresentation of the children’s data, the results were presented stratified by age. Another limitation was the lack of individual data regarding PCV13 and PPSV23 coverage, which could have played a role in the distribution dynamics of serotypes and clones. However, it was assumed that PPSV23 coverage remained stable and that PCV13 coverage increased during the study period. Another limitation is the use of bacterial culture as the sole criterion for definition of IPD with the potential to underestimate its burden. We and others have shown that the use of quantitative PCR (qPCR), for example, can improve sensitivity of detection of IPD (57, 58). Our objective was to compare the serotype and clonal distribution of S. pneumoniae over time with the inclusion of isolates from multiple health centers. While the use of bacterial culture is routinely performed in all these centers, the use of qPCR varies significantly: some centers had used PCR for more than 15 years, others had used it in the last 1 to 2 years, others used the technology only for meningitis, and others did not use it at all. To avoid unbiased results, only episodes detected by culture were included in this study. In addition, clonal type characterization is not possible in episodes that are detected only by PCR. Of note, the potential underestimation of the burden of IPD for both periods would be compared here.

In conclusion, a steady decrease of the number of IPD cases caused by vaccine serotypes indicates the importance and impact of systematic vaccination. Nevertheless, an increase of the nonvaccine serotypes highlights the importance of including more serotypes in future high-valent vaccines and considering better vaccination programs for older adults.

## MATERIALS AND METHODS

### Study setting and design.

We carried out a prospective study on invasive pneumococcal isolates received from 2013 to 2019 at the University Hospital Sant Joan de Déu in Esplugues, Barcelona. The Molecular Microbiology Department was nominated as the reference laboratory for molecular surveillance of IPD by the Catalan government in 2009. Our mission is to survey all the isolates causing IPD across the region. Although it is not compulsory for hospitals in Catalonia to send their samples for characterization, there are 25 health facilities that consistently send all isolates to the reference laboratory. For this study, only isolates sent by these 25 hospitals were analyzed.

In Catalonia, all hospital admissions by age and hospital are registered and publicly available with yearly updates (“Conjunt minim basic dades,” https://catsalut.gencat.cat). Similarly, the total population by age living in each township is recorded yearly in the public website (“Padró municipal d’habitants,” www.idescat.cat). Based on these data, in 2019 the estimated population was 7.67 million and the 25 health facilities served 62% of children younger than 5 years and 36% of the adult population.

Comparing the first year of the study (2013) versus the last one (2019), the reference population of the 25 health facilities varied between 217,452 and 249,975 in children of <5 years, 560,263 and 603,638 in 5- to 17-year-old children, 1,669,827 and 1,702,057 in 18- to 64-year-old adults, and 522,824 and 548,247 in ≥65-year-old adults (Table S1).

### Ethics statement.

This study was approved by the Ethics Committee of the Hospital Sant Joan de Déu (CEIm Fundació Sant Joan de Déu; Internal code, PIC-101-19).

No informed consent was requested for this study, as this is a molecular epidemiology surveillance-based study in which samples are duly anonymized.

### Definitions.

Invasive pneumococcal disease (IPD) was defined as the isolation of culturable S. pneumoniae from a patient’s normally sterile body site, such as blood, CSF, and pleural fluid. IPD was classified according to International Classification of Disease (ICD-10), specific for diseases caused by S. pneumoniae, including occult bacteremia/sepsis, meningitis, pneumonia, parapneumonic empyema, peritonitis, and arthritis. Recurrent IPD was defined as 2 or more episodes in the same individual at least 1 month apart.

The population study was divided into four groups according to their age: <5-year-old children, 5- to 17-year-old children, 18- to 64-year-old adults, and ≥65-year-old adults.

The study period included 3 years before the systematic introduction of the vaccine (2013 to 2015) and 3 years after (2017 to 2019). As the vaccine was introduced on the systematic vaccination schedule in July 2016, this year was not included in the study. The incidence rates of the first year of the study (2013) were considered the expected incidence rates (if no intervention had been done) and were used as reference.

### Microbiological identification and serotyping.

Isolates were identified at the hospital of origin by standard microbiological techniques such as Gram staining, culturing requirements, colony morphology, optochin sensitivity testing, and bile solubility test. After presumptive confirmation, the strains were sent to the Molecular Microbiology Department of the University Hospital of Sant Joan de Déu for epidemiological surveillance of S. pneumoniae. Upon reception of the isolates, multiplex PCR combined with fragment analysis and automated fluorescent capillary electrophoresis was performed to determine the identification of the capsular pneumococcal serotypes. This technique was implemented at the Molecular Microbiology Department in 2010 and allows the detection of 40 serotypes/serogroups: 1, 2, 3, 4, 5, 6A/6B, 6C, 7F/7A, 7C/(7B/40), 8, 9V/9A, 9N/9L, 10A, 10F/(10C/33C), 11A/11D/11F, 12F/(12A/44/46), 13, 14, 15A/15F, 15B/15C, 16F, 17F, 18/(18A/18B/18C/18F), 19A, 19F, 20, 21, 22F/22A, 23A, 23B, 23F, 24/(24A/24B/24F), 31, 33F/(33A/37), 34, 35A/(35C/42), 35B, 35F/47F, 38/25F, and (39) (59, 60). After rapid molecular serotyping, all strains were sent to the National Pneumococcus Reference Centre of Majadahonda, Madrid, Spain, for complete serotyping by the Quellung reaction, and these were considered the reference serotypes (61).

### Clonal analysis.

Multilocus sequence typing (MLST) was performed for all isolates for clonal assignment. MLST was performed by Sanger sequencing for all the strains isolated before 2019, as reported elsewhere (21), and by whole-genome sequencing (WGS) analysis for all strains isolated in 2019. WGS was carried out by MicrobesNG, a spinout company born in University of Birmingham, UK. The alleles obtained by Sanger were combined using the online software at the website http://pubmlst.org/spneumoniae/ to achieve the sequence type (ST). Analysis of WGS results was carried out by our department using the contigs provided by MicrobesNG. Contigs were analyzed with the online tools provided by the Center of Genomic Epidemiology (http://www.genomicepidemiology.org/) for allele assignation and ST assignment and the website https://pathogen.watch/ for capsular typing. Clonal complexes were assigned using eBURST (http://www.phyloviz.net/goeburst/) (62). STs that shared at least five out of seven allelic variants (double locus variants) were included in the same CC.

### Statistical analysis.

We used chi-square or Fisher’s exact tests to compare proportions. Incidence rates of IPD were calculated assuming constant population during a given year. We divided the study period in two categories, presystematic PCV13 vaccination period (PSVP) (2013 to 2015) and systematic PCV13 vaccination period (SVP) (2017 to 2019), for analyzing changes in specific serotypes and clones. Incidence rates were compared using the chi-square test with the rate ratio. Incidence rate ratios (IRRs) were estimated by dividing the incidence rate in the SVP period by the incidence rate in the PSVP period. We calculated 95% confidence intervals (CI), and 2-sided *P* values of <0.05 were considered to be statistically significant and were adjusted using false-discovery rate (FDR). Statistical analyses were performed using SPSS for Windows, version 17.0.
