# Supplementary material for: Serotypes and Clonal Composition of Streptococcus pneumoniae Isolates Causing IPD in Children and Adults in Catalonia before 2013 to 2015 and after 2017 to 2019 Systematic Introduction of PCV13
Source: Microbiol Spectr. 2021 Dec 8;9(3):e01150-21. doi: 10.1128/Spectrum.01150-21 (PMC8653838; doi:10.1128/Spectrum.01150-21)
Supplement: SUPPLEMENTAL FILE 2 — Supplemental material. Download SPECTRUM01150-21_Supp_1_seq7.pdf, PDF file, 0.2 MB [file spectrum01150-21_supp_1_seq7.pdf]

Table S1. Reference population by age group and years

Age groups	2013	2014	2015	2017	2018	2019
**<5**	249975	243813	236925	222294	229437	217452
**5-17**	560263	588346	594077	604352	638063	603638
**18-64**	1702057	1675222	1658254	1633929	1654821	1669827
**≥65**	522824	530099	535932	541939	550232	548247

Table S2. Incidence rates (per 100,000) of the top most prevalent serotypes by age group[c]

**Ages groups**	**Serotype**	**PSVP (IR*)**	**SVP (IR)**	***p-value***	***Adjusted p-value****
All ages	**8**	0.93	2.55	**<0.001**	**0.004**
	**3**	1.13	1.73	**<0.001**	**0.004**
	**12F**	0.93	1.10	0.123	0.189
	**15A**	0.32	0.67	**<0.001**	**0.004**
	**22F**	0.42	0.63	**0.026**	**0.058**
<5	**24F**	3.15	3.14	0.345	0.345
	**15A**	0.41	1.64	**0.026**	**0.058**
	**3**	0.55	1.64	**0.027**	**0.058**
	**19A**	1.23	1.49	0.340	0.345
	**15B/C**	0.41	1.49	**0.012**	**0.039**
	**8**	0.27	1.34	**0.027**	**0.058**
	**12F**	0.96	1.34	0.184	0.250
5-17	**8**	0.11	0.33	0.103	0.183
	**14**	0.11	0.22	0.247	0.284
	**18C**	0.06	0.16	0.202	0.250
	**3**	0	0.11	0.132	0.190
18-64	**8**	1.11	2.76	**<0.001**	**0.004**
	**3**	0.62	1.19	**0.001**	**0.004**
	**12F**	0.93	0.85	0.325	0.345
	**22F**	0.32	0.46	0.124	0.189
	**14**	0.52	0.42	0.252	0.284
>65	**3**	4.28	5.24	0.106	0.183
	**8**	1.57	4.88	**<0.001**	**0.004**
	**12F**	1.64	2.93	**0.007**	**0.026**
	**15A**	1.07	1.83	**0.04**	0.08
	**22F**	1.38	1.77	0.196	0.250

[c]IR, Incidence rate; PSVP, Pre-systematic vaccination period; SVP, Systematic vaccination period.

p<0.05 (Chi-square test).*_Adjusted p-values_ were calculated using FDR.

Table S3. Serotype variation along the years[d]

		PSVP (n=1048)			SVP (n=1255)		
**Serotypes**		**2013**	**2014**	**2015**	**2017**	**2018**	**2019**
**PCV13 serotypes**	***1***	42	29	15	2	1	2
	3	34	29	40	43	55	60
	4	5	0	5	6	6	3
	5	0	0	0	1	0	0
	6A	5	2	1	3	0	2
	6B	3	2	3	4	0	2
	7F	19	8	15	8	4	9
	9V	4	3	9	2	3	6
	14	22	34	34	12	16	18
	18C	2	2	7	3	6	6
	19A	21	18	14	20	13	13
	19F	6	8	4	7	8	7
	23F	4	2	3	5	2	2
**Non-PCV13 serotypes**	6C	8	11	12	7	7	5
	8	20	26	39	59	63	110
	9N	16	6	15	9	12	15
	10A	8	10	14	5	16	8
	11A	10	12	5	11	10	12
	12F	22	30	33	20	33	47
	15A	5	8	16	25	16	20
	16F	6	7	7	10	11	10
	22F	14	8	16	21	19	17
	23A	5	1	10	8	8	7
	23B	8	3	9	6	17	6
	24F	21	17	15	16	19	16
	31	3	6	7	6	8	9
	33F	5	5	7	9	9	14
	35B	2	5	8	6	12	5
	38	2	3	8	7	7	5
**Others (less prevalent serotypes)**	Others	20	15	25	28	32	37

[d]PSVP, Pre-systematic vaccination period; SVP, Systematic vaccination period, PCV13, 13-valent pneumococcal conjugate vaccine. Serotypes marked in cursive and bold are the ones included in the PPSV23 vaccine. Other less prevalent serotypes (n<30) include serotypes (2, 6E, 7B/C, 9VA, 11B, 13, 15B, 15C, 17F, 18A, 20, 21, 22A, 22FA, 24B, 27, 28A, 29, 34, 35F, 37, Non-typeable)

Table S4. Clonal distribution across the different age groups for both periods[e]

Age groups	CC/ST	No. PSVP	No. SVP	*p-value*	*Adjusted p-value**
<5	**CC156**	25	6	**<0.001**	**0.002**
	**CC230**	25	21	0.387	0.406
	**ST306**	11	0	**<0.001**	**0.002**
	**CC2372**	9	6	0.281	0.327
	**CC989**	7	7	0.436	0.436
	**CC97**	7	5	0.343	0.379
	**CC180**	3	6	0.1413	0.212
5-17	**ST306**	20	1	**<0.001**	**0.002**
	**CC53**	1	5	0.071	0.135
	**CC156**	1	4	0.121	0.212
18-64	**CC989**	46	37	0.181	0.248
	**ST306**	36	2	**<0.001**	**0.002**
	**CC53**	35	81	**<0.001**	**0.002**
	**CC156**	31	24	0.189	0.248
	**CC404**	18	48	**<0.001**	**0.002**
	**CC180**	17	46	**<0.001**	**0.002**
≥65	**CC156**	39	33	0.201	0.248
	**CC180**	37	63	**0.007**	**0.016**
	**CC260**	27	20	0.132	0.212
	**CC989**	26	42	**0.036**	**0.075**
	**CC53**	13	49	**<0.001**	**0.002**

[e]CC, Clonal Complex; ST, Sequence Type; PSVP, Pre-systematic vaccination period; SVP, Systematic vaccination period.

p<0.05 (Chi-square test). **Adjusted p-values* were calculated using FDR.

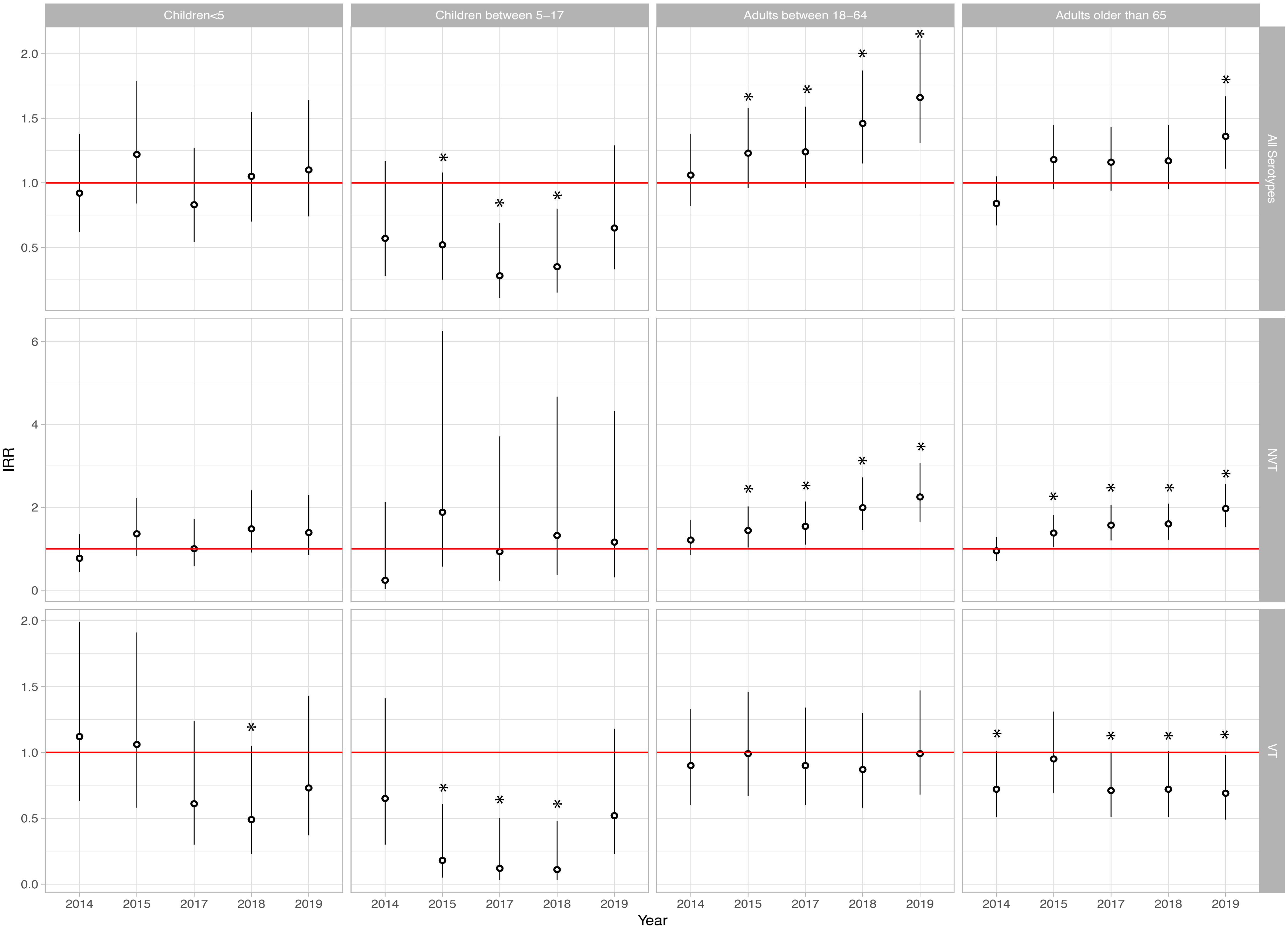

Figure S1. Post-PCV13 introduction invasive pneumococcal disease IRRs. The data are pooled IRRs. IRRs were estimated by comparing the observed incidence (in each of the years 2014, 2015, 2017, 2018 and 2019) to expected incidence (2013) for each age-serotype group. 95% CIs are represented on logarithmic sale by error bars.
